# Ectopic Permanent Molars: A Review

**DOI:** 10.3390/dj11090206

**Published:** 2023-08-29

**Authors:** Samah Alfuriji, Haifa Alamro, Jomanah Kentab, Lama Alosail, Linah Alali, Nada Altuwaijri, Rahaf Alalwan

**Affiliations:** 1 Department of Preventive Dental Sciences, College of Dentistry, King Saud bin Abdulaziz University for Health Sciences, P.O. Box 3660, Riyadh 11481, Saudi Arabia; amroh@ksau-hs.edu.sa; 2 King Abdullah International Medical Research Center, Ministry of National Guard-Health Affairs, P.O. Box 3660, Riyadh 11481, Saudi Arabia; 3 College of Dentistry, King Saud bin Abdulaziz University for Health Sciences, P.O. Box 3660, Riyadh 11481, Saudi Arabia; kentab109@ksau-hs.edu.sa (J.K.); alali202@ksau-hs.edu.sa (L.A.); altuwijri196@ksau-hs.edu.sa (N.A.); alalwan030@ksau-hs.edu.sa (R.A.); 4 Department of Periodontics, King Abdulaziz Medical City, Ministry of National Guard-Health Affairs, P.O. Box 22490, Riyadh 11426, Saudi Arabia; alosail007@ksau-hs.edu.sa

**Keywords:** ectopic, impacted, eruption, molar, early treatment, interceptive, tipping, wedging

## Abstract

Ectopic permanent molar is a condition in which the permanent tooth deviates from its normal path of eruption. The etiology of this eruption anomaly is multifactorial, with both general and local factors. The principal results suggest that a valid indicator of irreversible consequences is the degree of impaction of the first permanent molar. Self-correction is most common between the ages of 7 and 8, after which help may be required. Accordingly, early management can assist in preventing subsequent potential challenges that could interfere with maintaining a balanced occlusion. Several variables, including the degree of mesial tilting, the level of root resorption, and the condition of the second primary molar, may be crucial in choosing the most effective method of treatment. Interproximal wedging and distal tipping are the two basic therapeutic strategies for ectopic permanent molars. Additionally, the use of fixed or removable appliances might also be required. Delaying treatment until a later stage is not recommended because early diagnosis and treatment are essential for optimal management. This review aims to provide a comprehensive overview of ectopic permanent molars, including their prevalence, etiologic factors, self-correction rates, clinical implications for adjacent teeth, and various treatment techniques, that emphasizes the importance of early detection and intervention in the successful management of ectopic permanent molars. In addition, it highlights the importance of future research into the contributing variables of irreversible ectopic molar outcomes.

## 1. Introduction

An ectopic permanent molar is defined as a local disturbance in the developing dentition [1]. It can present as an abnormal alteration, deviation, or drifting of the tooth eruption pathway, which results in the first or second permanent molars erupting in contact with the distal undercut of the second deciduous molar or first permanent molar, respectively, instead of erupting into the normal occlusal plane [1]. Nikiforuk defined ectopic eruption as a condition in which the permanent teeth, because of a deficiency of growth in the jaw or segment of the jaw, assume a path of eruption that intercepts a primary tooth, causing its premature loss and producing a consequent malposition of the permanent tooth [1]. Throughout the literature, the terms ectopic eruption and impaction have been used interchangeably, and this is due to the fact that an ectopically erupting tooth might present with various degrees of impaction [2,3,4]. This can be seen in cases where the impaction (failure of eruption) of the first permanent molar takes place at the distal prominence of the second primary molar, and thus studies that have referred to the ectopic eruption as impaction were included. In this literature review, prevalence, classification, etiology and associated dentoskeletal features, diagnosis, complications and rationale for treatment, prognosis, treatment, and maintenance are discussed in detail. As the majority of the literature has focused on exploring the predictive factors and management techniques of ectopic molars, the present review intends to cover multiple aspects from previous studies to deliver a comprehensive review of ectopic permanent molars.

## 2. Prevalence

Most of the studies have focused more on the ectopic eruption of first permanent molars and fewer studies have reported the prevalence of ectopic second permanent molars [5].

The prevalence of ectopic first permanent molars ranges between 2 and 6%, with a higher incidence in the maxilla by 57.5% [6,7]. The total prevalence of disturbances in the eruption of second permanent molars was around 2.3% [5,8]. The incidence of ectopic second permanent molars is found to be 1.5%, and they occur more frequently in the mandible by 92% [5]. Most of the studies found no gender difference between males and females for both ectopic first and second molars [9]. However, a higher male incidence was reported among Turkish and Saudi populations [6,10].

## 3. Etiology and Associated Dentoskeletal Features

**Figure 1:**
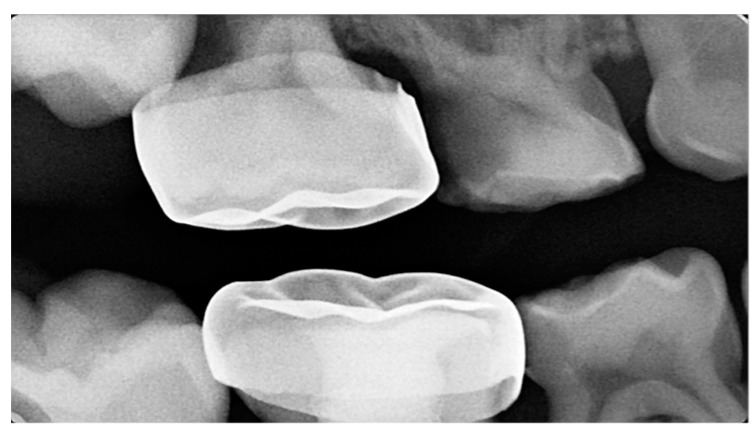
Ectopic eruption of the maxillary right first permanent molar due to improper marginal adaptation of the stainless-steel crown placed on the second primary molar. (Courtesy of Dr. H. Alamro.)

There is a general agreement among different authors throughout the literature regarding the etiology of ectopic eruption of permanent molars. It is considered a multifactorial pathological disorder with both general and local etiological factors, or a combination of both. General etiological factors include familial tendency and genetics [7,9]. Ectopic molar eruption has been reported among siblings with a 19.8% prevalence [9]. In addition, siblings are five times more likely than the overall population to be affected [11]. This familial tendency offers an opportunity for an early intervention of patients at risk [12]. In addition, there is an increased prevalence of ectopic molar eruption among cleft lip and palate patients by 25% and syndromic patients, like those with Turner syndrome and Apert syndrome, which indicates a possible genetic component of the problem [7,9,13]. In fact, patients with cleft lip and palate are four times more likely to have an ectopic permanent molar [7]. According to Ahiko et al., patients with Turner syndrome suffer from a retrognathic maxilla that leads to the ectopic eruption of the first permanent molar accompanied by resorption of the second primary molar [14]. Multiple local factors have been suggested, such as the imbalance between the mandibular growth in relation to the eruption of the first permanent molar. This would force a continued mesial inclination or an abnormal angulation of eruption of the molar and subsequently its entrapment under the distal bulge of the second primary molar [9,15,16,17]. Other local factors include maxillary hypoplasia and retrognathism, permanent molar macrodontia or delayed calcification, an unfavorable shape of the second primary molar crown, insufficient maxillary tuberosity growth, and crowding [9,15,16,17,18]. Moreover, an iatrogenic factor has been proven to cause an ectopic eruption of the first permanent molar. According to Croll et al., a lack of marginal adaptation of stainless-steel crowns placed on the second primary molars hinders the eruption of the first permanent molar. This failure to properly trim and contour the stainless-steel crown would impact adjacent teeth and cause malocclusion [19] (Figure 1).

Numerous studies have associated the ectopic eruption of first and second permanent molars with other dental disturbances [20,21,22]. Baccetti established a significant association among disturbances in the eruption of both first and second permanent molars and palatally displaced canines (30.76%) [20]. Another study found that 23% of patients had an ectopic maxillary first permanent molar in relation to the ectopic eruption of the maxillary canine [21]. This suggests that an ectopic eruption of maxillary molars and a pathological root resorption of the second primary molar leads to a higher risk of ectopic maxillary canines and resorption of maxillary permanent lateral and/or central incisors [21]. Additionally, another study also showed that ectopic maxillary canines appear more frequently in children with a primary diagnosis of maxillary first permanent molar eruption disturbances [22].

## 4. Classification

Throughout the literature, the ectopic eruption of the first permanent molar has many classifications based on several factors, such as spontaneous correction of its position, its effect on the second primary molar, and severity [23]. According to Young, ectopic eruption can be either reversible or irreversible [23]. He introduced the terms jump and hold. Jump, or self-correcting, is where the first permanent molar will spontaneously correct itself and erupt in its normal position [23]. In contrast, hold is the irreversible type and leads to a more permanent impaction of the molar with worse consequences [23]. Barberia-Leache et al. proposed another classification based mainly on the resorption severity effect on second primary molars (Table 1) [7,24]. Likewise, Harrison and Michal classified ectopic eruption according to impaction (Lock’s) severity, using the marginal ridge of the second primary molar as a reference on bitewing radiographs (Table 1) [25]. On the other hand, there is a lack of classification of ectopically erupting second and third molars throughout the literature. 

## 5. Diagnosis

Ectopic eruption is an asymptomatic anomaly that is usually diagnosed as an incidental finding during clinical examination or radiographic assessment. It should be suspected when there is greater than 6 months delay of eruption compared with its contralateral molar, when the first permanent molar is mesially angulated, or when asymmetry exists in the molar eruption [10,26]. Different radiographic techniques can be used to determine the ectopic eruption, such as bitewing radiographs, orthopantomography (OPG) radiographs, or cone-beam computed tomography (CBCT), with the latter being the most precise one among the techniques used [10]. In contrast, OPG radiographs only deliver no more than 10% of the radiation dose produced by CBCT and can be used as a reliable tool for measuring tooth angulation [26]. Thus, it is a viable tool in diagnosing ectopic eruption [26]. Early radiographic assessment should be a priority in 5–7-year-old children [9]. This helps to detect, intervene in, and avoid irreversible locking of the permanent molar [10]. Since the chances of self-correction are lower in children above the age of 7, a prompt diagnosis of such cases is critical to ameliorate the prognosis and palliate the consequences of the failure of eruption [10,26]. Otherwise, if not treated, it can incite various pathological conditions [26].

## 6. Complications and Rationale for Treatment

Untreated ectopic molars will result in a local disturbance of eruption and may lead to several harmful consequences, leading to impaired function and appearance [10]. Pain and infection around the second primary molar are often common complications resulting from an ectopically erupting first permanent molar [24]. As the severity of these complications increases, it leads to atypical pathological resorption and a premature loss of the second primary molar [10,24,26]. This results in mesial tipping, migration, and rotation of the first permanent molar, a deficiency in arch length in the affected quadrant, crowding of the corresponding arch segment, unilateral shifting of the maxillary molar towards a class II position, and the subsequent loss of space for the eruption of the second premolar, potentially leading to its impaction or delayed eruption [24,27]. Moreover, supraeruption of the opposing molar results in distortion of the curve of Spee and potentially leads to occlusal interference and multiple other orthodontic problems [10,24,26,27]. Similarly, an ectopically erupting second permanent molar might lead to an increased risk of root resorption, especially cervical root resorption of the first permanent molar, caries and periodontal problems, difficulties in treating deep bite, follicular cysts, malocclusion, pericoronal inflammation, and pain [8]. 

## 7. Prognosis

It has been documented that one-third of the cases remain locked after 7 years of age [26]. Therefore, through the ages of 7 to 8 years, an ectopic eruption of the first permanent molar is considered irreversibly locked. An additional important time milestone is when the maxillary molar reaches the level of the mandibular occlusal plane [10]. During the discussed period, an intervention is directed to establish proper vertical control and avoid supraeruption [10]. Various research has also explored some predictive factors for irreversible outcome. Dabbagh et al. analyzed the potential radiographic and clinical predictors for the irreversible outcome [28]. They reported that the extent of the impaction of the first permanent molar measured on bitewing radiographs was directly correlated with the irreversible consequence, and it was the most reliable predictor among the other assessed predictors, such as the degree of resorption, the amount of enamel ledge, the angulation of the molar, and the severity of the lock [26,28]. Also, it has been reported that bilateral ectopic eruption in males with an increased amount of impaction is positively associated with the irreversible type [28]. Bjerklin and Kurol had reported that the eruptive angulation and crown width of the first permanent molar were two critical factors in the outcome of ectopic eruptions [17]. However, a more recent study suggested that the eruptive angulation was interrelated with the onset of ectopic eruption; however, no significant difference was detected regarding the irreversible outcome [28]. Other possible reported factors that may lead to eruptive angulation are differences in bone-tooth size or some disturbances in the chronology of bone growth at the tuberosity area in relation to the calcification and eruption of the first permanent molar [10]. The genetic factor has also been associated with ectopic molars [10]. In addition, dental factors like caries or unfavorable morphology of the second primary molar might contribute to this eruption anomaly [26]. These conflicting results emphasize the importance of further investigations into the predictors for the irreversible outcome [26].

## 8. Treatment

Early intervention can help in preventing further complications that could affect the establishment of a balanced occlusion [29]. Therefore, delaying the treatment to a later stage is not advisable. The treatment of ectopic molars and the time of intervention depend on several factors [30]. Although previous studies have agreed that by the age of seven 66% of ectopic molars are self-corrected, others have stated that spontaneous eruption will not happen if the first permanent molar is not fully erupted at that age [4,29]. Therefore, the initiation of the treatment should be started immediately after the diagnosis is established. For those who are diagnosed before the age of 8, six months follow-up is recommended to allow time for self-improvement [7]. However, other studies have reported self-correction of ectopic maxillary first permanent molars after the age of 9 years in 71% of patients [28]. This might be a result of the resorption of the primary first molar and mesial drifting of the primary second molar, which allows the permanent first molar to move freely. This must be taken into consideration because unnecessary intervention might increase the risk of infection and accelerate the loss of the primary tooth. Moreover, overtreatment might be a waste of time and money for the patient and the practitioner. Thus, when the outcome is uncertain, delaying interceptive treatment is a possible option [28].

Several factors such as patient’s age, the severity of impaction, the degree of mesial tilting, the level of root resorption, and the status of the second primary molar could play an important role in determining the possible treatment options [8,25]. Generally, two main techniques are used for the management of ectopic permanent molars: interproximal wedging and distal tipping [23,30]. 

## 9. Ectopic First Permanent Molars

Based on the severity grading described by Barberia-Leache et al., in grade 1 ectopic molars the patient should be under observation and scheduled for a follow-up to allow spontaneous correction [7,24]. As the severity increases, active treatment is required and can be achieved through multiple techniques [7]. In grade 2 mild cases, interproximal wedging could be achieved using an elastic separator or Kesling separator [7], while in grade 3, the separation technique is not sufficient, so distal tipping of ectopic permanent molars using an active appliance is required [7]. This could be achieved using a fixed or removable appliance [7]. In grade 4, severe ectopic eruption can lead to severe root resorption of the second primary molar and space loss; therefore, extraction of the primary molar is recommended [23]. The consequences of second primary molar space loss can be avoided by placing a space maintainer or regainer, depending on the amount of space loss, to ensure the second premolar eruption is in its correct position [7]. In cases of congenitally missing second premolars, extracting the second primary molar and allowing the permanent molar to erupt mesially and close the space is suggested [7]. 

### 9.1. Interproximal Wedging 

**Figure 2:**
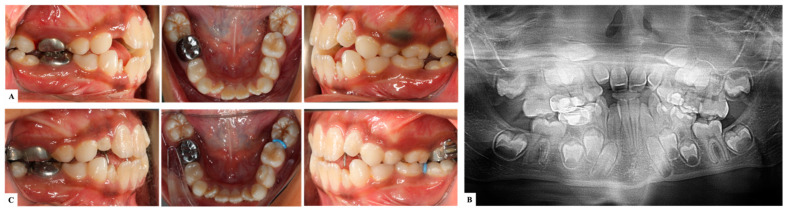
An 8-year-old patient with ectopic mandibular first permanent molars. (**A**). Pre-treatment lateral and occlusal clinical pictures showing the ectopic eruption of right first permanent molar against the stainless-steel crown of the primary second molar (iatrogenic), and ectopic eruption of the left first permanent molar against the crown of primary second molar. (**B**). Pre-treatment panoramic radiograph confirming the clinical findings. (**C**). Post-treatment lateral and occlusal clinical pictures showing the correction on the path of eruption of lower permanent molars using an elastic separator placed between the second primary molars and the first permanent molars replaced every two weeks. (Courtesy of Dr. S. Alfuriji.)

In cases where the first permanent molar is minimally or moderately impacted on the distal aspect of the second primary molar, interproximal wedging is chosen [7,30]. This option utilizes a wide range of separation techniques such as an elastic separator (Figure 2), soft brass wire, or a spring separator [7,30,31]. Disking the adjacent maxillary second primary molar using a 169 L carbide bur at high speed can also relieve the impaction of the permanent successor [30]. The soft brass wire separation technique was introduced by Levitas in 1964. It was frequently utilized as other techniques like elastic separators were not introduced yet. However, the softness of the wire was its main disadvantage and created difficulty in the passage between the second primary molar and the ectopic first permanent molar [32,33].

### 9.2. Distal Tipping

**Figure 3:**
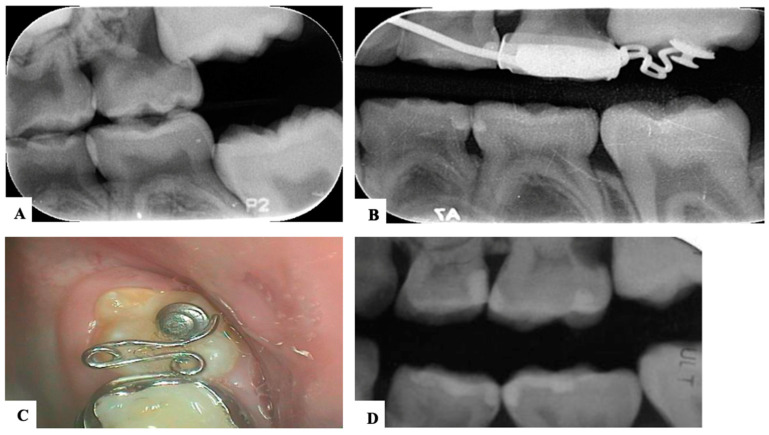
Ectopic maxillary first permanent molar with Halterman appliance to tip the first molar distally in a 5-year-old girl. (**A**). Pre-treatment radiograph showing maxillary left ectopic permanent molar. (**B**). Bitewing radiograph during appliance treatment. (**C**). Clinical photograph of the Halterman appliance 6 weeks later. (**D**). Post-treatment radiograph showing satisfactory eruption of the permanent molar. (Courtesy of Dr. Abu-Hussein M, permission granted.)

**Figure 4:**
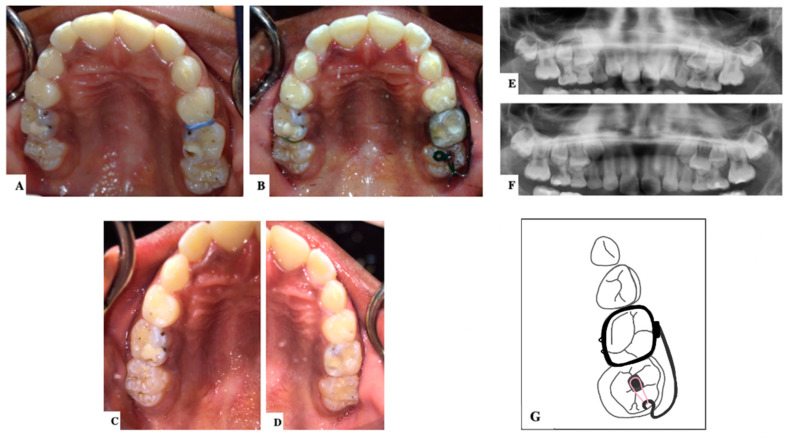
A case of an 8-year-old patient with a bilateral ectopic eruption of the maxillary first permanent molars. (**A**). Occlusal photograph before the treatment. (**B**). The right side showed a moderate degree of impaction and was treated using an elastic separator for distal wedging of #16. The left side showed a severe degree of impaction and was treated using a Halterman appliance for distal tipping of #26. (**C**,**D**). Occlusal photographs after the treatment, and the overall treatment duration was 3 months. (**E**). Panoramic radiograph before the treatment shows complete resorption of the disco-buccal root of the maxillary second primary molars. (**F**). Panoramic radiograph after the treatment. (**G**). Illustration of the Halterman appliance. (Courtesy of Dr. H. Alamro.) (Illustrations by Dr. Linah Alali.).

**Figure 5:**
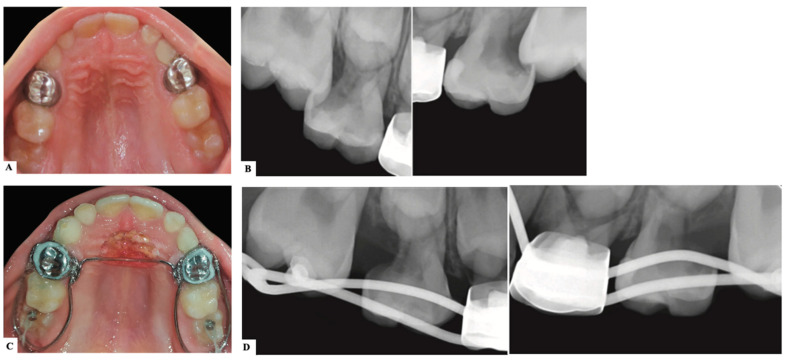
Bilateral ectopic maxillary first permanent molars with cemented modified Croll’s appliance to tip the first molars distally. (**A**). Pre-treatment clinical photograph showing bilateral ectopic eruption of the maxillary molars. (**B**). Pre-treatment radiograph. (**C**). Modified Croll’s appliance after cementation. (**D**). Post-treatment radiograph showing normal eruption of maxillary permanent molars. (Courtesy of Dr. Ambriss B, permission granted.)

In severe cases, distal tipping is the preferred option with or without the extraction of the second primary molar. Multiple fixed or removable appliances are used in the distal tipping technique [7,30]. Examples are a removable appliance with active cantilever springs [34], the Humphrey appliance, the Halterman appliance (Figure 3 and Figure 4) [35], sectioned wire with an open coil spring, a k-loop, and Croll’s appliance (Figure 5) [4,30]. Some authors used these appliances along with surgical exposure of the first permanent molar for better accessibility to button bonding [36].

## 10. Ectopic Second Permanent Molars

**Figure 6:**
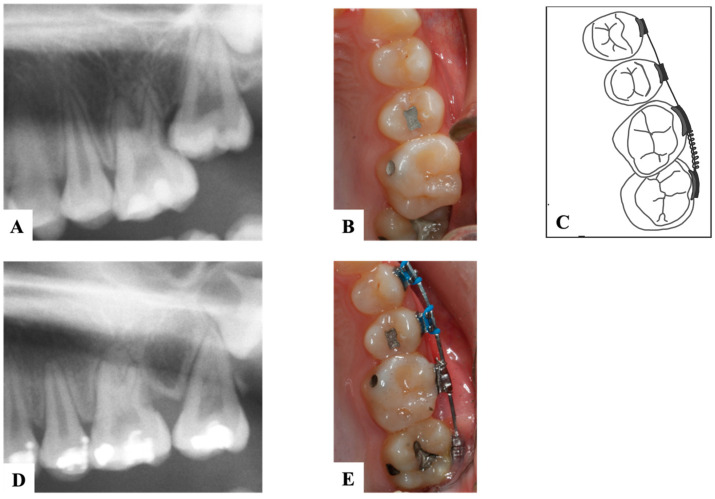
Ectopic maxillary left second permanent molar (#27) erupted against the first permanent molar (#26). (**A**). Pre-treatment radiograph showing the impaction of #27 against #26 causing a cervical root resorption on the distal surface of the first molar. (**B**). Occlusal photograph with partial eruption of #27. (**C**). Illustration of the appliance used to erupt #27, a fixed edgewise appliance with an open coil spring to tip and erupt #27 distally (illustrations by Dr. Linah Alali). (**D**). Progress radiograph with an erupted and aligned #27 with an obvious cervical root resorption on #26. (**E**). Occlusal photograph after alignment of #27. (Courtesy of Dr. S. Alfuriji.)

**Figure 7:**
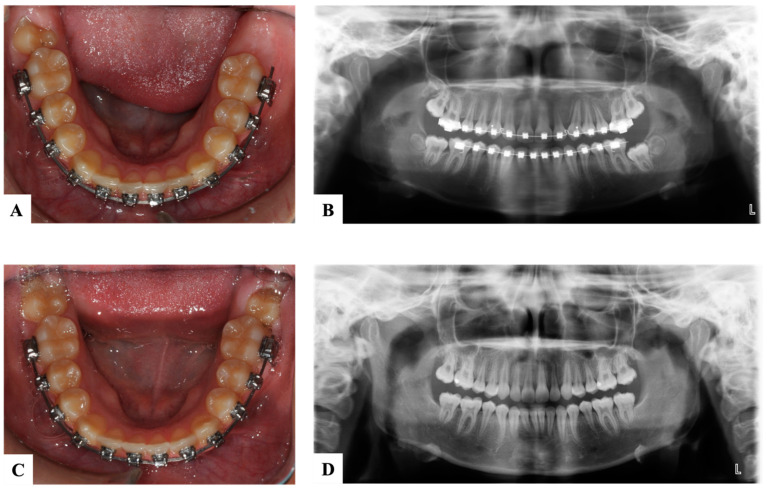
Ectopic mandibular left second permanent molar (#37) with impacted third molar (#38). (**A**,**B**). Occlusal photograph and panoramic radiograph before the treatment of the second molar (#37). (**C**). Occlusal photograph 11 days post-surgical uprighting of the second molar (#37) and extraction of third molar (#38). (**D**). Panoramic radiograph after the treatment. (Courtesy of Dr. S. Alfuriji.)

The treatment of ectopic second permanent molars differs from first molars in terms of the need for a combined orthodontic–surgical approach. In cases where the molar is partially impacted, distal tipping through fixed appliances can be used (Figure 6). In situations of complete impaction, surgical uprighting is the treatment of choice with or without removal of the third molars’ buds (Figure 7). The overall long-term prognosis of this procedure is good [37]. In cases where the second permanent molar is ectopically erupting, surgical exposure is performed followed by orthodontic treatment like using elastic separators [38]. Titanium screw implants can also be used as a source of anchorage to guide the second permanent molar into the correct position. If third molars are present, they should be extracted first before screw placement [39].

## 11. Novel Devices

**Figure 8:**
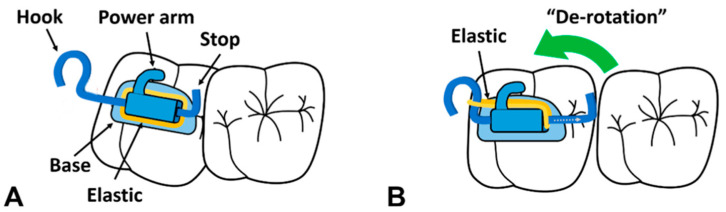
Design of the piston elastic. (**A**), The piston elastic is bonded on the occlusal surface of the ectopic molar, with its hook pointing distally; the elastic is placed as demonstrated. The angle between the straight part of the wire and the hook should be less than 90 to avoid the elastic slipping. (**B**). Device activation resulting in distal movement of the ectopic molar. (Courtesy of Dr. Kim IH, permission granted.)

**Figure 9:**
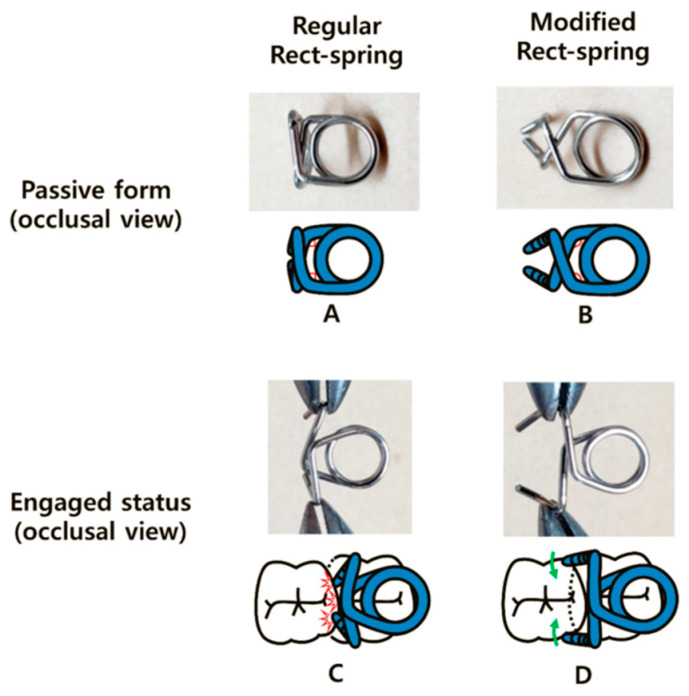
Comparison between the regular rect-spring and the modified rect-spring in the passive (**A**,**B**) and the engaged states (**C**,**D**). (Courtesy of Dr. Song MS, permission granted).

**Figure 10:**
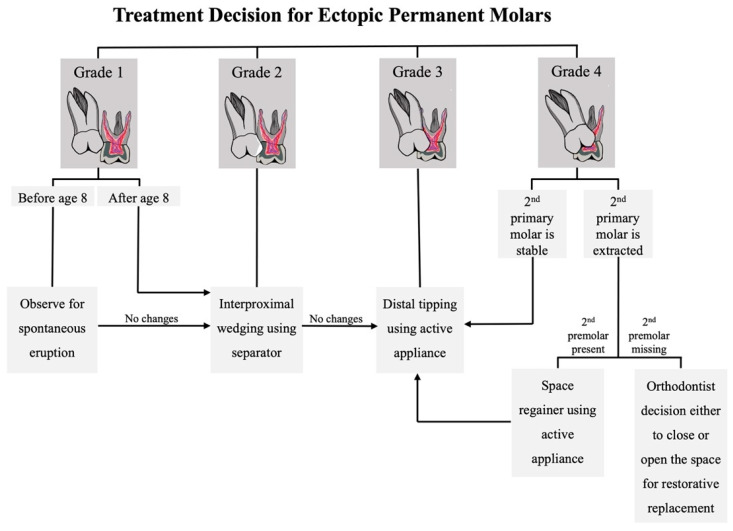
Treatment decision flowchart for ectopic first permanent molars.

Orthodontics is a developing field, with new devices and modifications of old ones emerging to achieve the best treatment outcomes. One of the latest introduced devices is the piston elastic, which functions through the distal tipping concept (Figure 8). Unlike other conventional methods, this device does not require impression taking or any laboratory-related work, which saves time and cost. However, the piston elastic is attached to the occlusal surface, and it can dislodge during mastication [40]. Other existing devices were modified to enhance their characteristics such as the rect-spring, which was introduced in 2014. The modification aimed to overcome the weak retention on mesially tilted molars (Figure 9). However, each device comes with its own limitations, and this device is not recommended for molars with severe tilting. Also, it can cause an anterior open bite due to unfavorable distal protrusion. Careful planning is crucial before deciding the correct treatment modality for any ectopic molar case [29,41]. A summary of different treatment modalities and a treatment decision flowchart are included (Table 2, Figure 10).

## 12. Maintenance

Relapse is a common occurrence after repositioning the first permanent molar [7]. A simple way to prevent this is by banding the second primary molar using a molar band that has a distal extension extended onto the occlusal surface [7]. The band is not to be removed until after the permanent molar has been sufficiently erupted to prevent relapse, and the patient should be recalled at 6–8-week intervals [7]. On the other hand, in cases where the second primary molar must be removed due to extensive root resorption or is lost prematurely, it is advised to plan for space maintenance or closure with the orthodontist in advance [7,42]. 

## 13. Conclusions

Ectopically erupting permanent molars present as an abnormal deviation of the natural tooth eruption pathway. It is a multifactorial pathological disorder with both general factors, such as familial tendencies and genetics, and local etiological factors, such as abnormal angulation of the permanent molar, maxillary hypoplasia, maxillary retrognathism, permanent molar macrodontia, and delayed permanent molar calcification. The diagnosis of ectopic permanent molars usually takes place as an incidental finding during clinical examination and radiographic assessment. Therefore, pediatric dentists and general practitioners need to be aware of the consequences of this condition and how to intervene early to avoid an irreversible locking of permanent molars that can lead to impaired function, impaired appearance, pain, infection, resorption to the surrounding tissues and structures, and, subsequently, the early loss of second primary molars, which would lead to crowding and multiple orthodontics problems. The treatment of ectopic molars and the time of intervention depend on several factors; however, there is a general agreement that the initiation of the treatment should start immediately after the diagnosis is obtained. Interproximal wedging and distal tipping are generally the two main techniques used for management. Treatment relapse can be prevented by banding the second primary molar until the first permanent one fully erupts and recalling the patient after 6–8 weeks. 

## Figures and Tables

**Table 1:** Classifications of ectopic first molars.

**Barberia-Leache ’s Classification** (Based on the effect on second primary molar) *					
**Grade 1** Mild	**Grade 2** Moderate		**Grade 3** Severe		**Grade 4** Very severe
Limited resorption to the cementum or with minimum dentine penetration	Resorption of the dentine without pulp exposure		Resorption of the distal root leading to pulp exposure		Resorption that affects the mesial root of the second primary molar
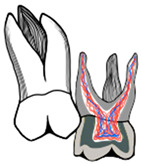	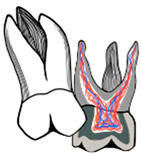		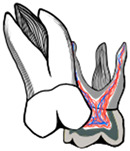		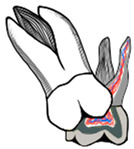
**Harrison and Michal’s Classification** (based on severity of the lock using bitewing radiograph) *					
**Normal**		**Minimal lock**		**Severe lock**	
No sign of impaction		Impacted less than half the width of the distal marginal ridge of the second primary molar		Impacted more than the width of the distal marginal ridge of the second primary molar	
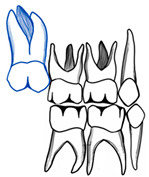		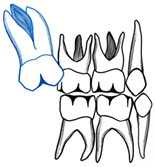		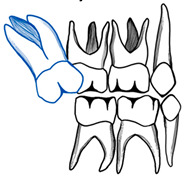	

* Illustrations by Dr. Linah Alali.

**Table 2:** Comparison between different treatment modalities.

Treatment Modalities	Severity of Impaction	Chair Side Time	Laboratory Work	Patient Discomfort	Cost	Treatment Duration
Elastic separator [35]	Mild to moderate	↓	✕	↑	↓	↓
Soft brass wire separator [37]	Mild to moderate	↓	✕	↑	↓	↓
Spring separator	Mild to moderate	↓	✕	↑	↓	↓
Humphrey appliance	Moderate to severe	↑	√	↓	↑	↓
Halterman appliance [35]	Moderate to severe	↑	√	↓	↑	↓
Croll’s appliance [4]	Moderate to severe	↑	√	↓	↑	↓
Fixed edgewise appliance	Moderate to severe	↑	✕	↓	↑	↓
Surgical uprighting	Severe	↑	✕	↑↑	↑↑	↑

## Data Availability

Not applicable.
